# Anti–SARS-CoV-2 and Autoantibody Profiles in the Cerebrospinal Fluid of 3 Teenaged Patients With COVID-19 and Subacute Neuropsychiatric Symptoms

**DOI:** 10.1001/jamaneurol.2021.3821

**Published:** 2021-10-25

**Authors:** Christopher M. Bartley, Claire Johns, Thomas T. Ngo, Ravi Dandekar, Rita L. Loudermilk, Bonny D. Alvarenga, Isobel A. Hawes, Colin R. Zamecnik, Kelsey C. Zorn, Jessa R. Alexander, Anne E. Wapniarski, Joseph L. DeRisi, Carla Francisco, Kendall B. Nash, Sharon O. Wietstock, Samuel J. Pleasure, Michael R. Wilson

**Affiliations:** 1 Hanna H. Gray Fellow, Howard Hughes Medical Institute, Chevy Chase, Maryland; 2 Weill Institute for Neurosciences, University of California, San Francisco; 3 Department of Psychiatry and Behavioral Sciences, University of California, San Francisco; 4 Department of Pediatrics, University of California, San Francisco; 5 Department of Neurology, University of California, San Francisco; 6 Biomedical Sciences Graduate Program, University of California, San Francisco; 7 Department of Biochemistry and Biophysics, University of California, San Francisco; 8 Chan Zuckerberg Biohub, San Francisco, California

## Abstract

**Importance:**

Neuropsychiatric manifestations of COVID-19 have been reported in the pediatric population.

**Objective:**

To determine whether anti–SARS-CoV-2 and autoreactive antibodies are present in the cerebrospinal fluid (CSF) of pediatric patients with COVID-19 and subacute neuropsychiatric dysfunction.

**Design, Setting, and Participants:**

This case series includes 3 patients with recent SARS-CoV-2 infection as confirmed by reverse transcriptase–polymerase chain reaction or IgG serology with recent exposure history who were hospitalized at the University of California, San Francisco Benioff Children’s Hospital and for whom a neurology consultation was requested over a 5-month period in 2020. During this period, 18 total children were hospitalized and tested positive for acute SARS-CoV-2 infection by reverse transcriptase–polymerase chain reaction or rapid antigen test.

**Main Outcomes and Measures:**

Detection and characterization of CSF anti–SARS-CoV-2 IgG and antineural antibodies.

**Results:**

Of 3 included teenaged patients, 2 patients had intrathecal anti–SARS-CoV-2 antibodies. CSF IgG from these 2 patients also indicated antineural autoantibodies on anatomic immunostaining. Autoantibodies targeting transcription factor 4 (TCF4) in 1 patient who appeared to have a robust response to immunotherapy were also validated.

**Conclusions and Relevance:**

Pediatric patients with COVID-19 and prominent subacute neuropsychiatric symptoms, ranging from severe anxiety to delusional psychosis, may have anti–SARS-CoV-2 and antineural antibodies in their CSF and may respond to immunotherapy.

## Key Points

**Question:**

Are anti–SARS-CoV-2 or antineural antibodies present in the cerebrospinal fluid of pediatric patients with COVID-19 and neuropsychiatric symptoms?

**Findings:**

In this case series of 3 pediatric patients with subacute neuropsychiatric impairment, 2 had intrathecal anti–SARS-CoV-2 antibodies as well as intrathecal antineural antibodies. Anti–transcription factor 4 (TCF4) autoantibodies in one patient who responded to immunotherapy were validated.

**Meaning:**

A subset of pediatric patients with COVID-19 and subacute neuropsychiatric symptoms have intrathecal antineural autoantibodies, suggesting central nervous system autoimmunity in pediatric patients with COVID-19 and recent neuropsychiatric symptoms.

## Teaser

This case series examines whether anti–SARS-CoV-2 and autoreactive antibodies are present in the cerebrospinal fluid (CSF) of pediatric patients with COVID-19 and subacute neuropsychiatric dysfunction.

## Introduction

More than 100 million people have been infected with SARS-CoV-2, including nearly 2 million children in the US.^1^ Although respiratory disease in pediatric COVID-19 is generally mild, parainfectious and postinfectious neurologic sequelae are increasingly recognized.^2,3^ These include encephalitis, seizures, aseptic meningitis, and confusion—found in about 20% of cases of multisystem inflammatory syndrome in children.^4^ Notably, rates of new and recurrent psychiatric illness are significantly increased in adults after SARS-CoV-2 infection compared with influenza and other respiratory infections.^5^

SARS-CoV-2 RNA is rarely detected in the cerebrospinal fluid (CSF) of patients with COVID-19, but intrathecal anti–SARS-CoV-2 antibodies have been reported,^6,7^ suggesting possible neuroinvasion. Although some neurologically impaired adults with COVID-19 have intrathecal antineural autoantibodies,^8^ to our knowledge, neither intrathecal anti–SARS-CoV-2 nor antineural antibodies have been reported in pediatric patients with COVID-19 and neuropsychiatric presentations.

## Methods

### Case Identification

Patients younger than 21 years who presented over 5 months in 2020 to the University of California, San Francisco (UCSF) Benioff Children’s Hospital with neuropsychiatric symptoms prompting a neurology consultation who also had evidence of a recent SARS-CoV-2 infection (positive findings on reverse transcriptase–polymerase chain reaction [RT-PCR] or serology with clinical history consistent with recent exposure) were considered for research enrollment. After parental consent, patients were enrolled in a UCSF research study for autoantibody detection in individuals with unexplained neuroinflammation. Over the same time period, there were 18 children hospitalized at UCSF Benioff Children’s Hospital with acute SARS-CoV-2 infection as documented by positive findings on a RT-PCR or rapid antigen test (hospital-wide serology results were unavailable). This study was approved by the University of California Human Research Protection Program and Institutional Review Board, and written informed consent was obtained from the parents of patients.

### Luminex-Based Anti–SARS-CoV-2 IgG Serology

To evaluate serological responses to SARS-CoV-2, peptide and whole-protein SARS-CoV-2 antigens were conjugated to Luminex beads. Sera (1:500 dilution) and CSF (1:20 dilution) were screened as previously described.^9^

### Anatomic Mouse Brain Immunostaining

As an initial screen for antineural autoantibodies, CSF immunostaining was performed as previously described^7^ but at a dilution of 1:4 for 48 hours at 4°C. For coimmunostaining, mouse brain tissue was immunostained with patient 1’s CSF (1:4 dilution) and anti-KIF21A (1:100 dilution) for 24 hours at 4°C. Further details on sequencing, assays, cloning, screening, and imaging can be found in the eMethods in the Supplement.

## Results

### Patient Presentations

All patients were hospitalized for subacute, functionally impairing behavioral changes and had SARS-CoV-2 infection by either nasopharyngeal swab RT-PCR or serology. Neuropsychiatric changes were concurrent with infection, determined by a positive RT-PCR result for patients 1 and 3 and by history and positive serology for patient 2 (Table).

**Table.** Clinical and Paraclinical Characteristics of Teenaged Patients With COVID-19 and Neuropsychiatric Symptoms

Characteristic	Patient 1	Patient 2	Patient 3
Age	Mid-teens	Mid-teens	Mid-teens
Hospital status	Transitional care unit (step-down unit)	Medical surgery ward	Pediatric intensive care unit
Ventilation	No	No	No
Blood laboratories at presentation			
Absolute neutrophil count, cells/μL	9320^a^	3590	12 810^a^
Absolute lymphocyte count, cells/μL	2330	1020	2130
Albumin, g/dL	4.1	4.3	5.6^a^
C-reactive protein, mg/dL	3.9^a^	0.6	27.6^a^
Erythrocyte sedimentation rate, mm/h	32	2	ND
Fibrinogen	ND	ND	ND
Procalcitonin, μg/L	ND	0.06	0.02
D-dimer, ng/mL	ND	ND	400
Ferritin, ng/mL	132^a^	ND	ND
Lactic acid dehydrogenase, U/L	252^a^	ND	ND
Interleukin 6	ND	ND	ND
Neurologic symptoms	Psychosis, delusions, mania, agitation, paranoia, disinhibited, poor attention, lability, and lower-extremity hyperreflexia	Psychosis, paranoia, agitation, feeling of impending doom, impulsivity, depression, suicidal ideation, bradyphrenia, and impaired working memory	Psychosis, insomnia, agitation, memory impairment, orofacial dyskinesias, catatonia, abulia, apraxia, and brisk reflexes
Estimated time from SARS-CoV-2 infection to neurologic symptoms	Concurrent	Concurrent	Concurrent
Estimated time from onset of neurologic symptoms to LP	14 d	Approximately 75 d to first LP; approximately 88 d to second LP	5 d
COVID-19 treatment	None	None	None
Neurologic treatment	IVIg, solumedrol, prednisone taper	Solumedrol, IVIg	Supportive care
Time from neurologic symptom onset to neurologic treatment	15 d	Approximately 77 d to solumedrol	NA
Outcome	Recovered	Partially recovered	Recovered
Comorbidities	Substance use disorder, PTSD, and anxiety	Tics and anxiety	None known
Neuroimaging	Brain MRI/MRA with and without contrast: few T2/FLAIR hyperintense foci in the white matter of bilateral frontal lobes	Brain MRI with and without contrast: unremarkable (once prior to solumedrol/IVIg, twice after solumedrol/IVIg); MRI of total spine without contrast (after solumedrol, before IVIg): No cord signal abnormality	Brain MRI/MRA with and without contrast: normal for age; MRI of cervical spine without contrast: normal for age
EEG	4-h Video EEG findings normal for age (obtained while taking valproate)	18 h (76 d After COVID-19 infection) and 15 h (88 d after COVID-19 infection) video EEGs: normal for age	15 h Video EEG: diffuse beta activity
Select additional negative testing	ENS2 and ENC2 panels	ENS2 and ENC2 panels	ENS2 and ENC2 panels
CSF profile			
WBC count, /uL	4	First LP, 1; second LP, 2; third LP, 1	1
Protein, mg/dL	117.0 (113.0 corrected)^b^	First LP, 48.0; second LP, 59.0; third LP, 80.0	19.0
Restricted OCBs	0	First LP, 0; second LP, ND; third LP, 0	3
IgG Index (Ref <0.7)	1.0	First LP, 0.6; second LP, ND; third LP, 0.6	0.6
SARS-CoV-2			
PCR			
NP	+	−	+
CSF	−	ND	−
IgG			
Plasma	+^c^	+^d^	ND
CSF	+^c^	+^c^	−^c^

Abbreviations: EEG, electroencephalography; FLAIR, fluid-attenuated inversion recovery; IgG, immunoglobulin G; IVIg, intravenous immunoglobulin; LP, lumbar puncture; MRA, magnetic resonance angiography; MRI, magnetic resonance imaging; NA, not applicable; ND, not determined; NP, nasopharyngeal; PCR, polymerase chain reaction; PTSD, posttraumatic stress disorder; WBC, white blood cell.

SI conversion factors: To convert neutrophils to ×10^9^/L, multiply by 0.001; lymphocytes to ×10^9^/L, multiply by 0.001; albumin to g/L, multiply by 10; C-reactive protein to mg/L, multiply by 10; D-dimer to nmol/L, multiply by 5.476; ferritin to μg/L, multiply by 1; WBC to ×10^9^/L, multiply by 0.001.

^a^ Elevated value.

^b^ Protein corrected for red blood cell count of 3075 cells/uL.

^c^ Research-based serology.

^d^ Clinical serology.

### Patient 1

A teenaged patient with a history of marijuana use, unspecified anxiety and depression, and limited psychiatric care presented with subacute social withdrawal, insomnia, erratic behavior, and paranoia-like fears. The patient tested positive for marijuana by urine toxicology. The patient tested positive for SARS-CoV-2 infection by RT-PCR of nasopharyngeal swab; however, the patient lacked respiratory symptoms. Approximately 2 weeks after presentation, the patient was discharged home taking risperidone and gabapentin. Shortly thereafter, the patient was readmitted because of signs of delusion and paranoia.

**Figure 1.**
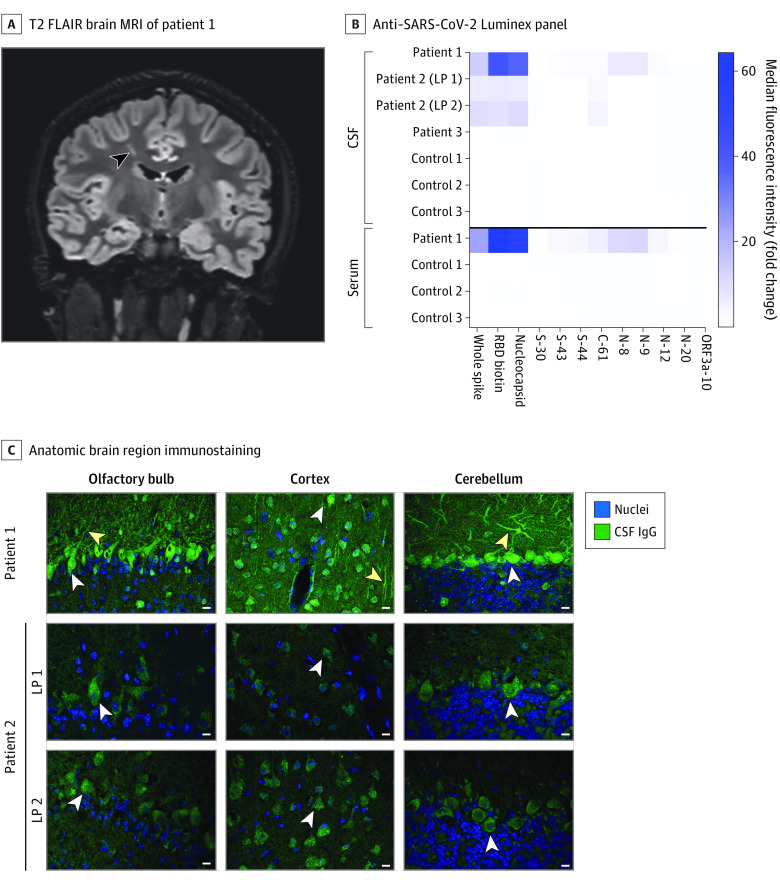
Anti–SARS-CoV-2 and Autoantibody Profiling A, Coronal 3-dimensional cube T2-weighted fluid-attenuated inversion recovery (FLAIR) brain magnetic resonance imaging (MRI) of patient 1. A linear hyperintense lesion in the right frontal centrum semiovale is noted (arrowhead). B, Cerebrospinal fluid (CSF) and sera from patients and controls were screened for anti–SARS-CoV-2 antibodies on a Luminex platform encoding whole-spike and nucleocapsid proteins, the spike receptor-binding domain (RBD), and spike, nucleocapsid, and ORF3a peptide antigens. The heatmap values represent the fold change of the median fluorescence intensity of averaged technical replicates for each biospecimen relative to negative controls. C, Select anatomic regions immunostained by patient 1’s CSF and CSF from patient 2’s first and second lumbar punctures (LPs) at a 1:4 dilution. White arrowheads in the olfactory bulb, cortex, and cerebellum indicate immunostained mitral cells, cortical neurons, and Purkinje cells, respectively. Yellow arrowheads indicate neuronal processes. Scale bars are 10 μM. IgG indicates immunoglobulin G.

The neurologic examination revealed asymmetric lower extremity hyperreflexia. The patient again tested positive for marijuana. Risperidone was replaced with olanzapine, then transitioned to valproate and lorazepam. CSF revealed elevated protein levels and an elevated IgG index (Table). Findings of autoimmune encephalopathy panels on CSF and serum were negative. Video electroencephalography (vEEG) findings were normal. Magnetic resonance imaging (MRI) of the brain showed nonspecific T2 and fluid-attenuated inversion recovery white matter hyperintensities in the frontal lobes without enhancement (Figure 1A). Given the patient’s subacute symptoms and CSF abnormalities in the context of COVID-19, there was concern for an inflammatory process, and the patient was treated with intravenous (IV) immunoglobulin, 2 g/kg, over 3 days, followed by IV methylprednisolone, 1 g, for 3 days and a prednisone taper starting at 60 mg per day. Five days after initiating immunotherapy (day 20 after symptom onset) the patient had more organized thoughts, decreased paranoia, and improved insight. More than 1 month after symptom onset, the patient’s delusions and hyperreflexia had resolved; however, the patient’s lability persisted, and they were discharged taking valproate.

### Patient 2

A teenaged patient with a history of unspecified anxiety and motor tics endorsed foggy brain. The patient’s father was diagnosed with COVID-19 by nasopharyngeal RT-PCR that week. Five days later, the patient developed fever and respiratory symptoms, which resolved without treatment. SARS-CoV-2 testing was not performed. Concurrently, the patient then developed word-finding difficulties, impaired concentration, and difficulty completing homework. The patient had insomnia and mood lability characterized by sobbing and elation. Over 6 weeks, the patient experienced internal preoccupation, aggression, and suicidal ideation and did not respond to aripiprazole or risperidone and were prescribed lithium. Approximately 10 weeks after symptom onset, the patient was admitted to the hospital.

Findings of neurologic examination were notable for bradyphrenia and impaired working memory. Illicit substances were not detected by urine toxicology. Prior SARS-CoV-2 infection was confirmed by positive SARS-CoV-2 IgG serology. CSF protein levels were elevated, and findings of MRI of the brain with and without gadolinium were normal (Table). vEEG findings were normal. The patient’s working memory and bradyphrenia improved after receiving IV methylprednisolone, 1 g per day, for 5 days, and they were discharged home taking lithium and risperidone.

The patient was readmitted 6 days later for extreme anxiety, passive suicidal ideation, sadness, insomnia, and aggression after discontinuing risperidone and starting sertraline. On examination, subtle akathisia and chorea were noted; however, repeated neuroimaging and autoimmune studies were unrevealing. A repeated lumbar puncture showed persistently elevated CSF protein levels (Table). Given the parainfectious onset of psychiatric symptoms, chorea, and the elevated CSF protein levels, the patient received an empirical trial of IV immunoglobulin, 2 g/kg, over 3 days and was discharged taking quetiapine and lithium.

Six months later, although improved from initial presentation, the patient required academic accommodations and continued to endorse forgetfulness and attention difficulties. The patient’s chronic tics and anxiety were unchanged. Findings of repeated contrast brain MRI were normal, and a third lumbar puncture again revealed persistently elevated CSF protein level (Table).

### Patient 3

A teenaged patient with no known psychiatric history presented to the emergency department after 4 days of odd behavior, including repetitive behaviors, anorexia, and insomnia. On admission, the patient was tachypneic, but their oxygen saturation was 100%. Findings of the patient’s SARS-CoV-2 nasopharyngeal RT-PCR were positive. The patient reported taking an unknown substance a few days prior to presentation, but findings of a comprehensive urine toxicology were negative. The patient had an elevated white blood cell count, creatine kinase level, and C-reactive protein level (Table). They exhibited ideomotor apraxia, abulia, disorganized behavior, agitation, and diffusely brisk reflexes. The patient was treated with olanzapine and lorazepam, and their CSF had 3 unique oligoclonal bands, but findings of autoantibody testing were negative. Although findings of MRI of the brain with and without gadolinium were unremarkable, vEEG revealed diffuse beta activity. Olanzapine and lorazepam were discontinued, and the patient’s mental status gradually improved over the next week. The patient was discharged at their normal neurologic baseline without psychiatric medications.

### Antibody Studies

#### SARS-CoV-2 Luminex Serology

Patients 1 and 2 had CSF IgG against SARS-CoV-2 spike protein, receptor-binding domain, and nucleocapsid protein (Figure 1B). Serum from patient 1 showed the same anti–SARS-CoV-2 IgG profile as the CSF.

#### Anatomic Immunostaining

Patients 1 and 2—those harboring intrathecal SARS-CoV-2 IgG—immunostained a diversity of brain regions at 1:4 dilution. Patient 1’s CSF strongly immunostained mitral cells in the olfactory bulb, cortex, cerebellar Purkinje cells, and brainstem as well as other regions (Figure 1C; eFigure 1 in the Supplement). Patient 2’s CSF from both time points immunostained the same anatomic regions as patient 1 (Figure 1C; eFigure 1 in the Supplement). Patient 3 showed minimal staining of the hilus at 1:4 and no staining elsewhere (eFigure 1 in the Supplement).

#### Human Phage Immunoprecipitation Sequencing

**Figure 2.**
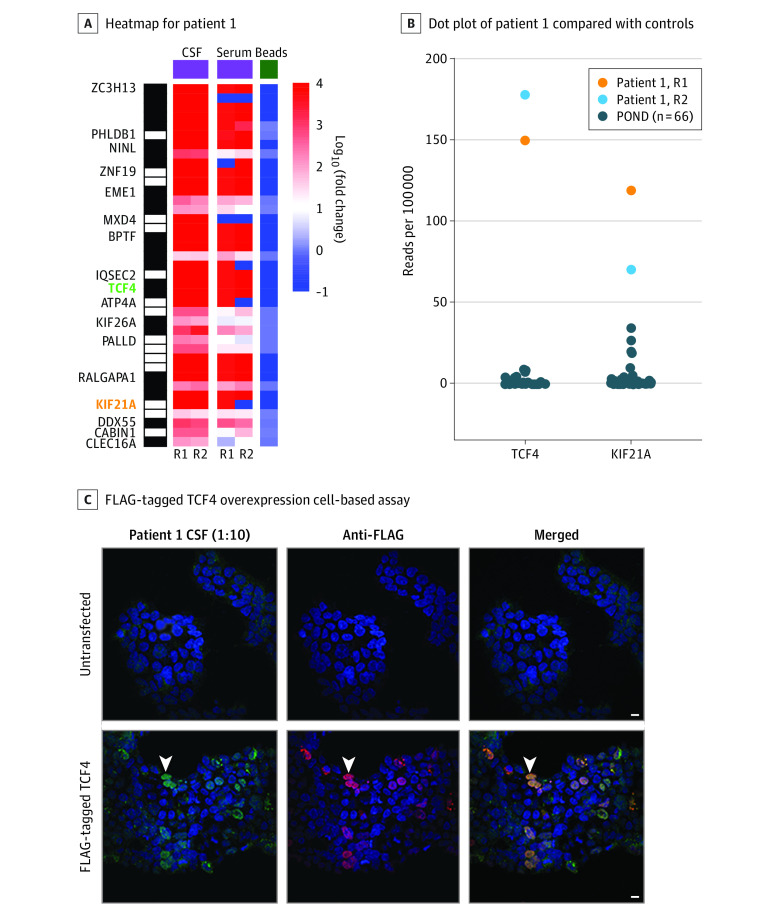
Anti–SARS-CoV-2 and Autoantibody Profiling A, Heatmap of human phage display immunoprecipitation sequencing data for patient 1’s cerebrospinal fluid (CSF) and serum. TCF4 and KIF21A are highlighted in green and orange, respectively. Values represent log_10_(fold change) relative to the mean reads per 100 000 for bead controls. The black and white barcode on the left indicates rows of individual peptides that correspond to a given protein. B, Dot plot of patient 1’s CSF TCF4 and KIF21A peptide human phage display immunoprecipitation sequencing read counts compared with CSF from 66 pediatric patients with other neurologic diseases (POND). C, HEK 293 cell overexpression cell-based assay. Untransfected cells or cells transfected with FLAG-tagged TCF4 were immunostained with CSF (1:10) and a rabbit anti-FLAG antibody. Cells were then counterstained with anti–human IgG 488 (green) and anti–rabbit IgG 594 (red). Arrowheads indicate an example of a TCF4-overexpressing cell that was immunostained by CSF IgG. Scale bars are 10 μM. R1 and R2 indicate technical replicates 1 and 2, respectively.

Patient 1’s CSF enriched 17 candidate autoantigens relative to bead-only controls, including the brain-enriched proteins kinesin-like protein KIF21A (KIF21A; gene ID, 55605) and transcription factor 4 (TCF4; gene ID, 6925) (Figure 2A). Patient 1’s CSF also enriched TCF4 and KIF21A peptides relative to CSF samples from pediatric patients with other neurologic diseases (Figure 2B). For patient 2’s CSF, 18 candidate autoantigens were enriched across both CSF samples (eFigure 2 in the Supplement). Patient 3’s CSF did not enrich any candidate autoantigens.

#### Anatomic Coimmunostaining

We coimmunostained mouse brain tissue with patient 1’s CSF and a commercial antibody to KIF21A and found that the immunostaining substantially overlapped (eFigure 3 in the Supplement). However, there was not sufficient remaining CSF for definitive validation.

#### Overexpression Cell-Based Assay

To validate TCF4, we immunostained FLAG-TCF4-overexpressing HEK 293 cells with patient 1’s CSF and an anti-FLAG antibody. CSF immunostained and colocalized with FLAG-TCF4 but did not stain untransfected cells (Figure 2C).

## Discussion

We profiled intrathecal antibodies in 3 teenaged patients with subacute neuropsychiatric symptoms after SARS-CoV-2 infection, none of whom met criteria for multisystem inflammatory syndrome in children. All 3 had abnormal CSF with restricted oligoclonal bands, elevated protein levels, and/or an elevated IgG index. On research testing, patients 1 and 2 had intrathecal anti–SARS-CoV-2 IgG. CSF IgG from these 2 patients also immunostained mouse brain tissue, indicating the presence of antineural autoantibodies. Likewise, patients 1 and 2 enriched a diverse set of candidate autoantigens by human phage immunoprecipitation sequencing, while patient 3 neither appreciably immunostained nor enriched candidates by human phage immunoprecipitation sequencing.

The outcomes for these patients differed. Patient 1 was unresponsive to psychiatric medications, and their symptoms remitted after immunotherapy. Patient 2 experienced a modest response to immunotherapy, but 6 months later, the patient continued to have impaired mood and cognitive symptoms. Patient 3’s symptoms remitted after 4 days of treatment with lorazepam and olanzapine without immunotherapy.

Overall, these findings indicate that severe neuropsychiatric symptoms can occur in the setting of pediatric COVID-19, including patients who lack many of the cardinal systemic features. Like the adult COVID-19 population, measures of CSF inflammation may be subtle or absent. Nevertheless, we found that 2 patients had intrathecal SARS-CoV-2 and antineural antibodies. In one patient, CSF immunostaining overlapped with commercial KIF21A, an axonal kinesin implicated in congenital fibrosis of extraocular muscles and abnormal brain development.^10^ In that same patient, we validated autoantibodies against TCF4, a gene that has been associated with psychiatric disorders, including schizophrenia, and is responsible for the neurodevelopmental disorder Pitt-Hopkins syndrome.^11^ These data highlight the possibility of SARS-CoV-2 neuroinvasion and/or CNS autoimmunity in pediatric patients with COVID-19 and neuropsychiatric symptoms.

### Limitations

This study has limitations. This study was an uncontrolled convenience sample with a small sample size. Patients 1 and 2 had preexisting neuropsychiatric symptoms. We cannot rule out that patients 1 and 2 improved independent of immunotherapy, owing either to concurrent treatment with psychiatric medications or to the passage of time. It is unclear whether these antineural antibodies are pathogenic or specific to these clinical syndromes.

## Conclusions

A subset of pediatric patients with COVID-19 and neuropsychiatric symptoms harbor intrathecal anti–SARS-CoV-2 and antineural autoantibodies. These data motivate a systematic study of humoral immunity in the CSF of pediatric patients with COVID-19 and neuropsychiatric involvement and the potential for immunotherapy in some.

## Notes

Supplement.
eMethods.
**eFigure 1.** Extended overview of anatomic immunostaining. **eFigure 2.** Phage display immunoprecipitation sequencing results for cases 1 and 2. **eFigure 3.** Coimmunostaining of case 1 CSF and commercial anti-KIF21A.
eReferences.
