# Supplementary material for: Anti–SARS-CoV-2 and Autoantibody Profiles in the Cerebrospinal Fluid of 3 Teenaged Patients With COVID-19 and Subacute Neuropsychiatric Symptoms
Source: JAMA Neurol. 2021 Oct 25;78(12):1–6. doi: 10.1001/jamaneurol.2021.3821 (PMC8546622; doi:10.1001/jamaneurol.2021.3821)
Supplement: Supplement. — eMethods. eFigure 1. Extended overview of anatomic immunostaining. eFigure 2. Phage display immunoprecipitation sequencing results for cases 1 and 2. eFigure 3. Coimmunostaining of case 1 CSF and commercial anti-KIF21A. eReferences. [file jamaneurol-e213821-s001.pdf]

Supplemental Online Content

Bartley CM, Johns C, Ngo TT, et al. Anti–SARS-CoV-2 and autoantibody profiles in the cerebrospinal fluid of 3 teenaged patients with COVID-19 with subacute neuropsychiatric symptoms. *JAMA Neurol*. Published online October 25, 2021.
doi:10.1001/jamaneurol.2021.3821

eMethods.

eFigure 1. Extended overview of anatomic immunostaining

eFigure 2. Phage display immunoprecipitation sequencing results for cases 1 and 2

eFigure 3. Coimmunostaining of case 1 CSF and commercial anti-KIF21A

eReferences.

This supplementary material has been provided by the authors to give readers additional information about their work.

© 2021 Bartley CM et al. *JAMA Neurology*.

eMethods.

Human phage display immunoprecipitation sequencing (PhIP-Seq)[1]: Our PhIP-Seq library encodes the entire human proteome as 49 amino acid peptides that overlap by 25 amino acids[2,3]. Incubation of the PhIP-Seq library with human IgG allows for isolation and enrichment of putative autoantigens. We screened patient CSF (all cases) and sera (case 1 only) by PhIP-Seq in technical replicate as previously described[4] and identified candidates using a Z-score threshold of 3 in both technical replicates relative to protein A/G beads only.

HEK 293 Overexpression Assays

Vector	Additional Details
pENTR223-TCF4	Human ORFeome clone 2069
pDEST26-C-FLAG	Addgene, Plasmid #79725
pDEST26-TCF4-C-FLAG	Human ORFeome clone 2069

Cloning

The entry vector containing an *attL*-flanked gene insert encoding *transcription factor 4* (pENTR223-TCF4, UCSF ORFeome) was gateway cloned into an *attR*-destination vector, pDEST26-C-FLAG (Addgene, Plasmid #79725) with Gateway™ LR Clonase™ II Enzyme mix (ThermoFisher, 11791020). pDEST26-C-FLAG was a gift from Rita Shiang (Addgene plasmid # 79275 ; http://n2t.net/addgene:79275 ; RRID: Addgene_79725). DH5α competent cells (ThermoFisher, Cat. #18265017) were transformed using the gateway product mix,

© 2021 Bartley CM et al. *JAMA Neurology*.

plated onto Ampicllin-100 LB plates. pDEST26-TCF4-C-FLAG plasmids were obtained from culture using Macherey-*Nagel*´s NucleoSpin® Plasmid Transfection-grade miniprep kit (Cat. #740490), then sequenced.

HEK293 Cell-Based Assay Autoantigen Screening

HEK293 cells were plated onto 10mm poly-d-lysine coated (50µg/mL) coverslips in 24-well plates. 293 cells were transfected overnight with pDEST26-TCF4-C-FLAG plasmids using Lipofectamine 3000 (ThermoFisher). The following day, after two rinses with ice cold 1X PBS, pDEST26-TCF4-C-FLAG transfected cells were fixed were 4% PFA for 10 minutes. The fixed cells were rinsed with PBS, blocked with 5% lamb serum in PBS, and permeabilized for 30 minutes using with blocking buffer containing 0.5% Triton.

pDEST26-TCF4-C-FLAG overexpressing HEK293 cells were stained overnight with anti-FLAG antibody at 1:1000 and CSF at 1:4 in 5% blocking buffer. The cells were rinsed with PBS four times and stained with Alexa Fluor secondaries at a 1:1000 dilution in 5% blocking buffer, pDEST26-TCF4-C-FLAG cells with anti-human 488 and anti-rabbit 594. Nuclei were stained with DAPI at 1:2000 in PBS for 5 minutes. Stained slides were then mounted onto microscope slides with Prolong Glass Antifade (ThermoFisher, Cat. P36980).

© 2021 Bartley CM et al. *JAMA Neurology*.

**Primary Antibodies**			
**Name**	**Host Species**	**Vendor (Catalog No.)**	**Assay (Concentration)**
Anti-FLAG antibody (D6W5B)	Rabbit	Cell Signaling Technology *(#14793*)	ICC-IF (1:1000)
Anti-KIF21A	Rabbit	LSBio (LS-B1542)	IHC-IF (1:100)
**Secondary Antibodies**			
**Fluorophore**	**Specifications**	**Vendor (Catalog No.)**	**Assay (Concentration)**
Alexa Fluor 488	AffiniPure Donkey Anti-Human IgG (H+L)	Jackson ImmunoResearch (709-545-149)	IHC-IF (1:500)<br>ICC-IF (1:1000)
Alexa Fluor 594	AffiniPure Donkey Anti-Rabbit IgG (H+L)	Jackson ImmunoResearch (711-585-152)	IHC-IF (1:500)<br>ICC-IF (1:1000)

.

Imaging

Slides were imaged at 60X at the UCSF Nikon Imaging Center using a Nikon CSU-W1 spinning disk confocal microscope, equipped with an Andor Zyla sCMOS camera.

© 2021 Bartley CM et al. *JAMA Neurology*.

eFigure 1. Extended overview of anatomic immunostaining

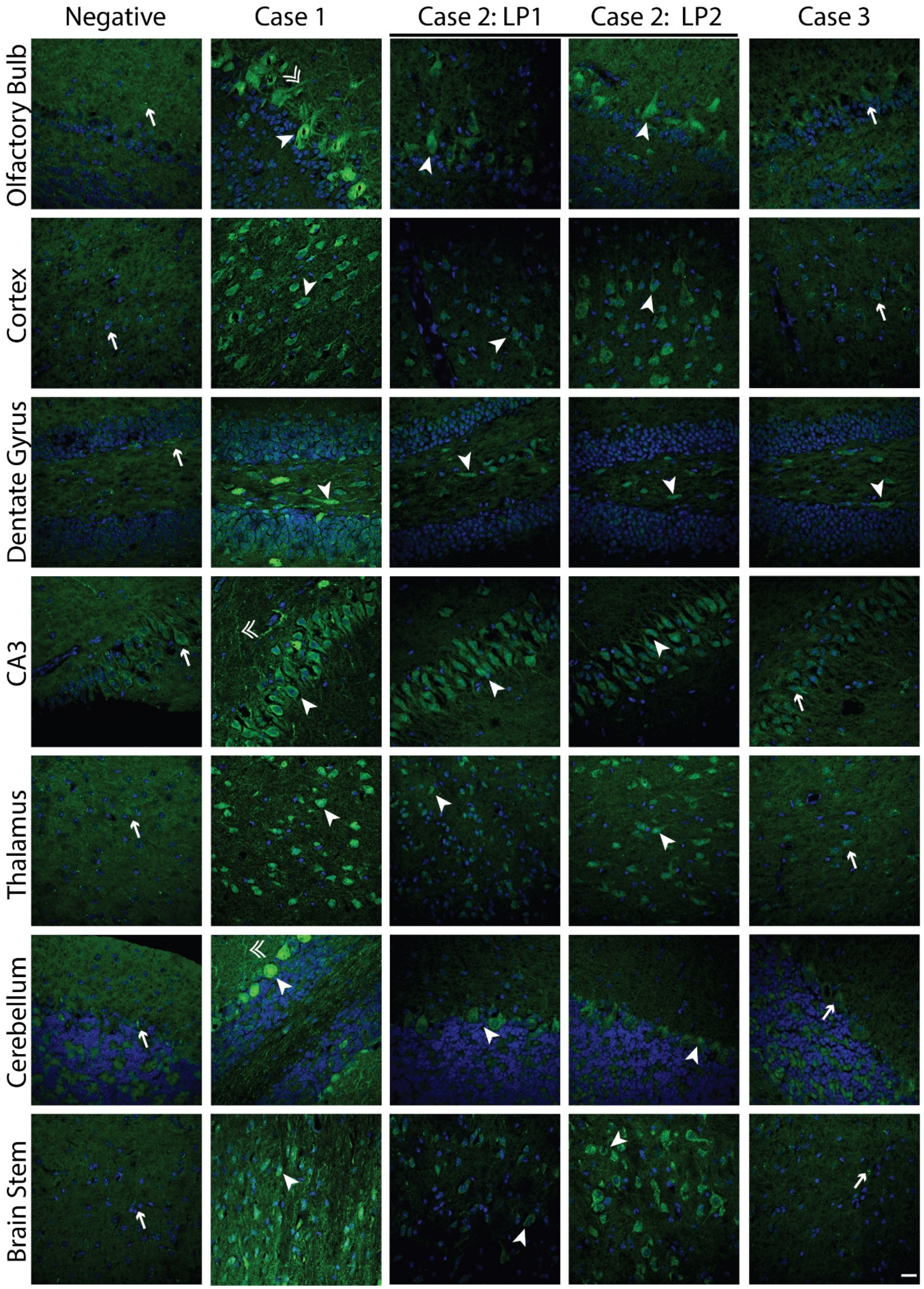

© 2021 Bartley CM et al. *JAMA Neurology*.

Sagittal sections of adult mouse brain tissue were immunostained with CSF from cases 1, 2, and 3 at a 1:4 dilution. Single arrows highlight non-specific or indeterminant immunostaining. Arrowheads indicate immunostained cell bodies. Double arrowheads highlight immunostained cell processes. Case 2 immunostaining in olfactory bulb, cortex, thalamus, and brain stem was visually more prominent at the second time point. All images were acquired at 60x magnification. The scale bar in the lower right panel is 20µm.

© 2021 Bartley CM et al. *JAMA Neurology*.

eFigure 2. Phage display immunoprecipitation sequencing results for cases 1 and 2

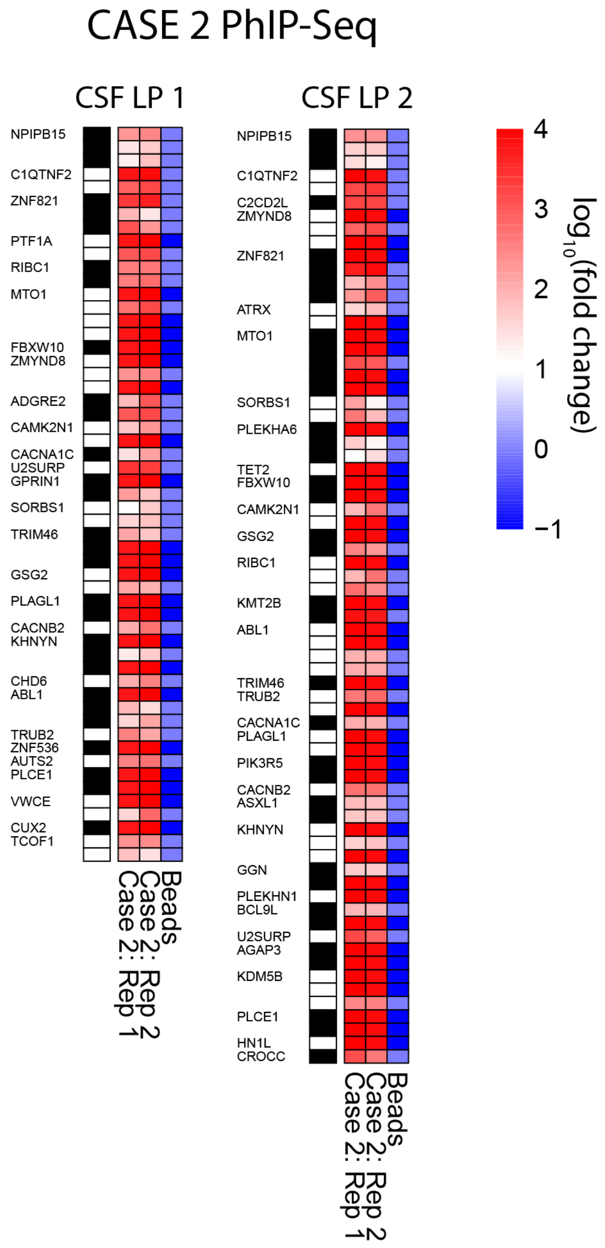

In technical replicate (Rep 1/Rep 2), biospecimens were screened for autoantibodies against a whole human peptidome T7 bacteriophage display immunoprecipitation sequencing platform (PhIP-Seq). Heatmap values represent the log(fold change) over bead controls. For each heatmap, official gene identifiers are shown on the left. The white and black barcode indicates sets of peptides (rows), that map to a given gene/protein on the left. Case 3 did not enrich any candidate peptides above background and is not shown.

© 2021 Bartley CM et al. *JAMA Neurology*.

eFigure 3. Coimmunostaining of case 1 CSF and commercial anti-KIF21A

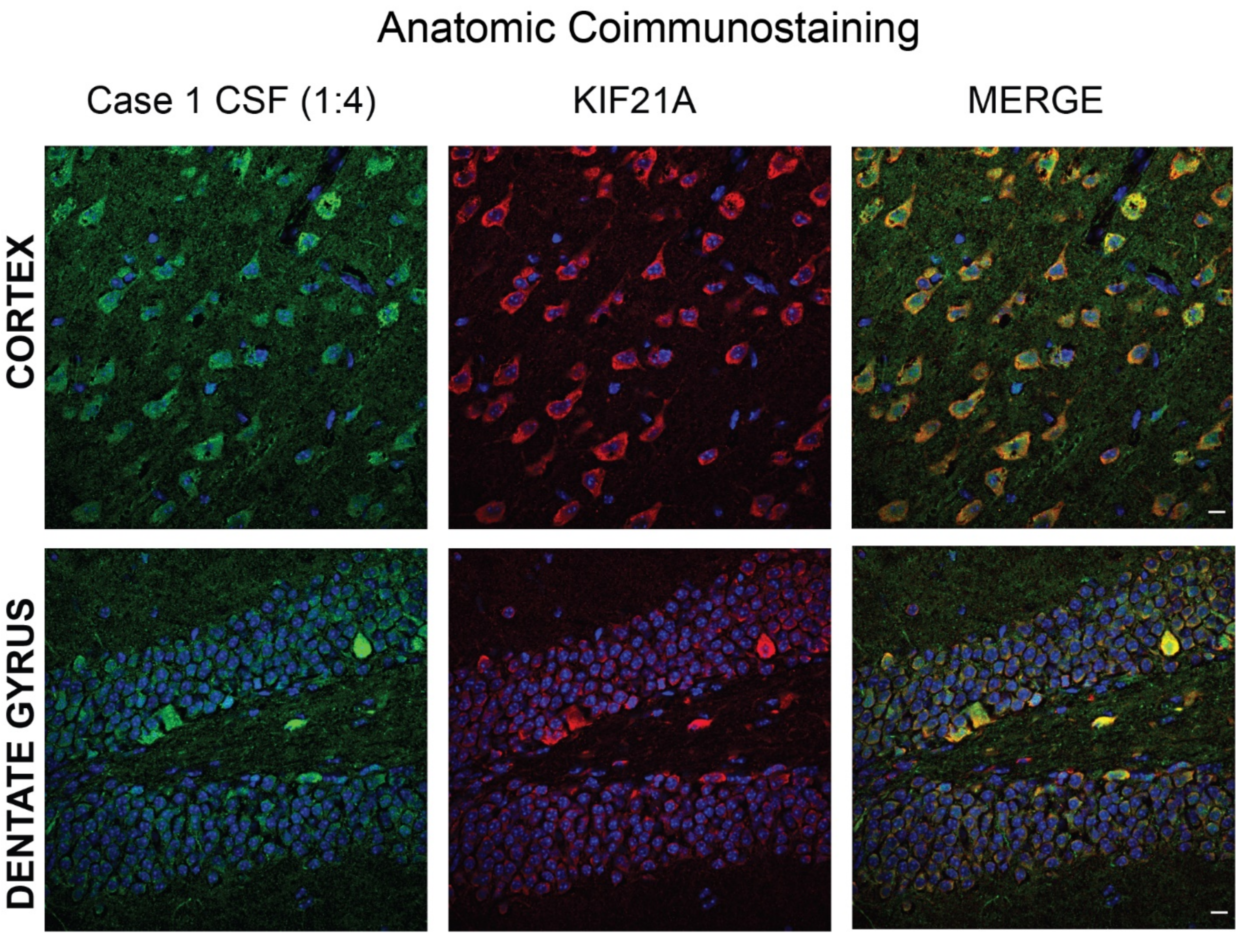

Anatomic coimmunostaining shows colocalization of case 1 CSF IgG and commercial anti-KIF21A in the cortex and dentate gyrus of the hippocampus. Scale bars = 10 µM.

© 2021 Bartley CM et al. *JAMA Neurology*.

eReferences.

1. Larman HB, Zhao Z, Laserson U, et al. Autoantigen discovery with a synthetic human peptidome. Nat Biotechnol. 2011;29(6):535-541. Medline:21602805 doi:10.1038/nbt.1856

2. Mandel-Brehm C, Dubey D, Kryzer TJ, et al. Kelch-like protein 11 antibodies in seminoma-associated paraneoplastic encephalitis. N Engl J Med. 2019;381(1):47-54. Medline:31269365 doi:10.1056/NEJMoa1816721

3. O'Donovan B, Mandel-Brehm C, Vazquez SE, et al. High-resolution epitope mapping of anti-Hu and anti-Yo autoimmunity by programmable phage display. Brain Commun. 2020;2(2):fcaa059.

4. Schubert RD, Hawes IA, Ramachandran PS, et al. Pan-viral serology implicates enteroviruses in acute flaccid myelitis. Nat Med. 2019;25(11):1748-1752. Medline:31636453 doi:10.1038/s41591-019-0613-1

© 2021 Bartley CM et al. *JAMA Neurology*.
